# How the Effect of Maternal Age on the Risk of Childhood Leukemia Changed over Time in Sweden, 1960–2004

**DOI:** 10.1289/ehp.11938

**Published:** 2008-11-07

**Authors:** Milena Maria Maule, Loredana Vizzini, Kamila Czene, Olof Akre, Lorenzo Richiardi

**Affiliations:** 1 Cancer Epidemiology Unit, Center for Experimental Research and Medical Studies and Center for Oncologic Prevention in Piedmont, University of Turin, Turin, Italy; 2 Department of Medical Epidemiology and Biostatistics, Karolinska Institutet, Stockholm, Sweden; 3 Clinical Epidemiology Unit, Department of Medicine, Karolinska University Hospital, Karolinska Institutet, Stockholm, Sweden

**Keywords:** childhood leukemia, epidemiology, maternal age, rate ratios, time trends

## Abstract

**Background:**

Previous studies on the association between maternal age and risk of childhood leukemia found inconsistent results.

**Objectives:**

We aimed to assess whether there is an association between maternal age and risk of childhood leukemia and whether such an association is modified by maternal year of birth.

**Methods:**

By linking nationwide Swedish registers, we analyzed leukemia incidence among all children between 1 and 5 years of age born between 1960 and 1999. We estimated incidence time trends by child year of birth (overall and stratified by maternal age) and incidence rate ratios (RRs) for maternal age groups stratified by maternal birth cohort. We tested the interaction between maternal age and child year of birth through the likelihood ratio test between nested Poisson regression models.

**Results:**

We observed 1,562 leukemia cases. The overall annual percent change (APC) was 1.00 [95% confidence interval (CI), 0.51 to 1.49]. Stratifying by maternal age classes, APCs decreased from 1.66 (0.68 to 2.65) for mothers ≤ 24 years to 0.23 (−0.93 to 1.40) for mothers ≥ 35 years at delivery. RRs for children born to the oldest with respect to the youngest mothers were 2.42 (1.31 to 4.67), 1.68 (1.00 to 2.72), 1.34 (0.87 to 2.01), and 0.87 (0.46–1.54) for mothers born in 1930–1934, 1940–1944, 1950–1954, and 1960–1964, respectively.

**Conclusions:**

Childhood leukemia risk increased with maternal age for mothers born in the past, whereas maternal age had no effect on this risk for mothers born more recently. This finding may explain the inconsistency of previous studies and suggests that leukemia risk may be related to an environmental factor to which women’s exposure has changed over time.

Leukemias are the most frequent childhood malignancies, representing approximately one-third of all malignant neoplasms occurring in children younger than 15 years of age both in Europe (Coebergh et al. 2006) and in the United States (Smith et al. 1999). Childhood leukemia is a collection of biologically diverse diseases whose largest subgroup is acute lymphocytic leukemia [ALL; 81% of cases in Europe (Coebergh et al. 2006) and approximately 75% in the United States (Smith et al. 1999)]. To date, the etiology of childhood leukemia remains largely unclear, and several pathways to the development of one of its forms have been suggested (Dickinson 2005). In particular, the carcinogenic process leading to childhood leukemia is thought to start with the acquirement of genetic translocations during fetal life and to progress toward overt disease through a postnatal final event induced by environmental exposures (Greaves 2006; McNally and Parker 2006).

Parental age could theoretically increase the risk of childhood leukemia if, for instance, genetic hits accumulate in the germ cells (Walter et al. 2003). Several studies have investigated the effect of parental age (Cnattingius et al. 1995; Dockerty et al. 1999, 2001; Hemminki et al. 1999; Hjalgrim et al. 2004; Jourdan-Da Silva et al. 2004; Kaye et al. 1991; Ma et al. 2005; Manning and Carroll 1957; Maule et al. 2007a, 2007b; McKinney et al. 1999; Mogren et al. 1999; Murray et al. 2002; Reynolds et al. 2002; Roman et al. 1997, 2005; Ross et al. 1997; Savitz and Ananth 1994; Schuz et al. 1999; Shaw et al. 1984; Shu et al. 1988; van Steensel-Moll et al. 1985; Westergaard et al. 1997; Yip et al. 2006; Zack et al. 1991) on the risk of childhood leukemia, but results have been inconsistent. It is noteworthy that older studies more often found a positive association (Manning and Carroll 1957), whereas the most recent and largest studies did not (Hjalgrim et al. 2004; Roman et al. 2005; Schuz et al. 1999), with some exceptions (Dockerty et al. 2001; Reynolds et al. 2002; Yip et al. 2006).

Findings in Sweden (Cnattingius et al. 1995; Hemminki et al. 1999; Mogren et al. 1999; Yip et al. 2006; Zack et al. 1991) and the Nordic countries (Hjalgrim et al. 2004) have not been consistent, although the studies used partially overlapping data.

Three case–control studies including children born in Sweden in 1973–1984 (Zack et al. 1991) and 1973–1989 (Cnattingius et al. 1995) and children diagnosed in Denmark, Sweden, Norway, and Iceland in 1984–1999 (Hjalgrim et al. 2004) found no association between maternal age and childhood leukemia risk. On the other hand, three Swedish population-based cohort studies including children diagnosed in 1958–1994 (Mogren et al. 1999), 1960–1994 (Hemminki et al. 1999), and 1961–2000 (Yip et al. 2006) showed that older maternal age was associated with an increased risk of leukemia, especially in the age group 0–4 years (Hemminki et al. 1999; Yip et al. 2006).

In this study we analyzed data on all children 1–5 years of age who were born in Sweden between 1960 and 1999 to investigate whether childhood leukemia incidence trends vary with maternal age at delivery and, if such an association exists, whether it is different in different maternal birth cohorts.

## Materials and Methods

### Study population

The study population was identified through a linkage of several Swedish data sources using the national registration number (NRN). All Swedish residents alive in 1947 onward have been assigned a 10-digit NRN (date of birth plus a 4-digit code containing information on sex and county of birth), which is a unique personal identifier referred to in all medical records and official registries. Through the use of the NRN, it is possible to link information from several databases together.

Since 1958, all newly diagnosed malignant tumors in Sweden must be reported to the National Cancer Registry by the physician who makes the diagnosis as well as the pathologist or cytologist who confirms it (The National Board of Health and Welfare, Swedish Cancer Registry: http://www.social-styrelsen.se/en/Statistics/statsbysubject/Cancer+Registry.htm). We used the seventh revision of the *International Classification of Diseases* (ICD-7) (World Health Organization 1957) to identify leukemia (ICD-7 code 204-9) in the present study.

In 2000, Statistics Sweden began a linkage between several data sources from the national registration and created the Swedish Multi-Generation Register, which contains information on the parents of all individuals in Sweden born in 1932 onward and survived until 1961. Using this register, it is thus possible to identify siblings and offspring of each index person through the parents. The completeness of the register increases rapidly with increasing year of birth (Statistics Sweden 2007). For those born in 1935, 80% of the parents can be identified, and parent information is virtually complete for those born 1945 onward (Statistics Sweden 2007). Adoption or other nonbiologic relations are flagged in the register.

By linking the registers above, we were able to obtain data on the cumulative occurrence of leukemia between 1 and 5 years of age specifically for each combination of birth year and maternal age at birth. At the time of linkage, cancer register data were available through 2004. We therefore limited the analyses to all children born from 1960 through 1999 who had complete follow-up between 1 and 5 years of age.

The study was approved by the Stockholm Regional Ethics Committee.

### Statistical analysis

We used a nonparametric generalized linear mixed model with a second-order autoregressive error component to obtain smoothed incidence time trends by birth cohort of the children (Breslow and Clayton 1993; Maule et al. 2006).

We calculated the annual percent change (APC) by fitting a least-squares regression line to the natural logarithm of the leukemia incidence rates, using the child year of birth as a regressor variable. The calculation was performed both overall and stratifying by maternal age group to assess whether incidence trends varied with maternal age at delivery.

We tested the interaction between maternal age and child year of birth through the likelihood ratio test by fitting two nested Poisson regression models (one including maternal age and child year of birth and the other including both terms and their interaction).

We calculated rate ratios (RRs) and their 95% confidence intervals (CIs) by median-unbiased estimation (Rothman and Greenland 1998). The Breslow–Day test (Breslow and Day 1987) was used to estimate RR trends by maternal age for different maternal birth cohorts to assess if the association between childhood leukemia incidence and maternal age at delivery was different in different maternal birth cohorts.

All statistical tests were two-sided.

## Results

**Figure 1:**
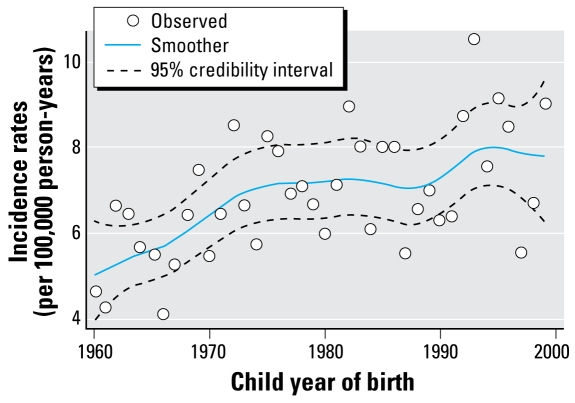
Observed and expected incidence rates of leukemia at 1–5 years of age, by child year of birth. Expected values (smoother) and 95% Bayesian credibility intervals (dashed lines) were obtained using a generalized linear mixed model. Data from Swedish Cancer Registry, 1960–2004.

We observed 1,562 cases of leukemia. As shown in previous Swedish studies (Yip et al. 2006), leukemia incidence rates increased over the study period (Figure 1). The estimated APC for children born between 1960 and 1999 was 1.00 (95% CI, 0.51–1.49). The APC in incidence of childhood leukemia was modified by maternal age (*p*-value for interaction = 0.021). Specifically, the increasing incidence trend was limited to children born to mothers < 30 years of age (Table 1).

**Figure 2:**
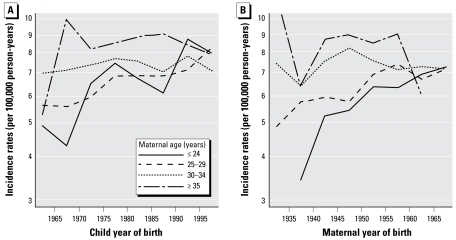
Incidence rates of childhood leukemia (per 100,000 person-years) at ages 1–5 years over child year of birth (*A*) and over maternal year of birth (*B*) for maternal age classes. Data from Swedish Cancer Registry, 1960–2004.

Figure 2 shows incidence rates of childhood leukemia (per 100,000 person-years) over the child year of birth (left) and the maternal year of birth (right) for the same maternal age groups. As noted above, incidence rates increase with time for children born to younger mothers (≤ 24 and 25–29 years of age), until they reach the level of those born to older mothers (30–34 and ≥ 35 years of age) (7–8 cases per 100,000 person-years). The figure shows that all curves level off (that is, incidence rates become similar for mothers of all ages) approximately for children born after 1990 or for mothers born after 1955.

The observed number of leukemia cases and the corresponding incidence rates by maternal year of birth and maternal age are shown in Table 2. Corresponding RRs with 95% CIs are shown in Table 3. Until the maternal birth cohort 1955–1959, RRs of leukemia in the offspring increased with increasing maternal age, whereas no effect of maternal age was observed in offspring of women born in 1960 onward.

## Discussion

We found that leukemia incidence for children 1–5 years of age born to mothers > 30 years of age has been fairly constant in Sweden since 1960. On the other hand, in the same period, increasing time trends are apparent for children born to younger mothers, with leukemia incidence almost doubling for children born in 1960 compared with those born in 1999. In other words, children with older mothers had higher risk of leukemia than children with younger mothers in the past, whereas in more recent times the same risk is shared by children with mothers of all ages.

The major strength of our study is the length of the time series of leukemia cases, essential to investigate temporal changes of incidence and effect modification with maternal age. The Swedish Cancer Registry covers the whole country and provides complete and high-quality population-based data (Mattsson and Wallgren 1984). This has allowed the investigation of the role of maternal age in childhood leukemogenesis in a population that has been quite ethnically homogeneous during the study period. Furthermore, being the linked product of the Swedish Cancer Registry and the Multi-Generation Register, the database analyzed is not affected by selection or ascertainment biases.

Nevertheless, some possible limitations must be considered in interpreting our results. Separate analyses of leukemia subtypes were not possible in our study. Reliable diagnoses of ALL are available only starting from the 1970s (Pui 1995). However, temporal behavior of childhood leukemia in the age range considered in our study is likely to be dominated by ALL, which accounts for approximately 90% of all leukemias in children 1–5 years of age (Pizzo and Poplack 2006). To verify this assumption, we calculated RRs by maternal age groups for the two most recent maternal birth cohorts (1960–1964 and 1965–1969) specifically for ALL. Estimated coefficients for the linear trend differed only marginally from those calculated for all leukemias (data not shown).

Another relevant limitation of our work was the absence of information on potential confounders or effect modifiers, such as birth order and paternal age. A large U.K. study including more than 3,000 children born and diagnosed with ALL in 1968–1986 (Dockerty et al. 2001) found that low parity and high maternal age independently increase ALL risk in the child. According to these results, because older mothers are more often primiparous and have fewer children in recent years than in the past, we would have expected to observe higher risk of leukemia in children born to older mothers in the most recent periods. Because our findings go in the opposite direction (higher risk for children with older mothers only in the past, similar incidence rates for children with mothers of all ages in recent times), confounding by parity cannot explain but possibly dilutes the observed increasing ALL risk with maternal age in older periods.

We did not investigate the role of paternal age. Maternal and paternal ages are strongly correlated, so their effects are difficult to disentangle. In the United Kingdom, Dockerty et al. (2001) found that ALL risk was higher for children with older mothers and fathers but obtained little improvement in the model’s fit when paternal age was included in a model that already included maternal age, and a greater impact in the reverse procedure. Similarly, in Sweden, Yip et al. (2006) found an effect of both maternal and paternal ages on the risk of childhood leukemia (0–4 years of age) but maternal age effect remained stronger after mutual adjustment. Finally, in Italy, we found that older maternal age was associated with ALL risk, regardless of adjustment for paternal age (Maule et al. 2007b).

Previous findings on the effect of maternal age on the risk of childhood leukemia often have disagreed even when the studies were conducted in the same geographic area and for overlapping periods of time. Of six epidemiologic studies conducted in Sweden (Cnattingius et al. 1995; Hemminki et al. 1999; Hjalgrim et al. 2004; Mogren et al. 1999; Yip et al. 2006; Zack et al. 1991), three case–control studies did not find an effect (Cnattingius et al. 1995; Hjalgrim et al. 2004; Zack et al. 1991), and three cohort studies did (Hemminki et al. 1999; Mogren et al. 1999; Yip et al. 2006). Our findings suggest that results of these previous studies are not really conflicting, but that an effect of maternal age had existed in Sweden and has changed in time, so that some studies captured it and some others did not. Other similarly inconsistent results in studies conducted in other countries (Ma et al. 2005; Reynolds et al. 2002; Shaw et al. 1984) might be the result of a similar phenomenon, which could have occurred concomitantly or at a different time or with a different time scale.

In addition to resolving apparently inconsistent results, the findings of our study provide some clues in the search of potential risk factors for leukemia onset. The statistically significant interaction between child year of birth and maternal age suggests that the association between maternal age and childhood leukemia risk is unlikely to be the result of the accumulation of random events or damages in maternal germ cell DNA. Should this be the case, differences in leukemia incidence by maternal age would remain fairly constant in time rather than disappearing in more recent birth cohorts, unless we hypothesize that the speed at which DNA damages accumulate has accelerated in recent times. It seems more likely that maternal age is an indicator of the level of the child’s exposure to some (unknown) environmental exposures either during fetal life or in the first months after birth. To explain the change of risk over time, such an exposure should have occurred later in life for women born in the past and more rapidly, or at an earlier age for women born in more recent times.

In conclusion, we found that the risk of childhood leukemia in Sweden increased with maternal age for children born in the past, but became rather stable across maternal age groups for children born in more recent years. This finding may resolve the inconsistency of previous studies conducted in Sweden, and possibly elsewhere, and suggests that if the triggering event of childhood leukemia onset is an environmental factor to which mothers are exposed, then their exposure modality must have changed over time, starting off earlier in life in more recent years.

## Figures and Tables

**Table 1:** Annual percentage change and 95% CI in leukemia incidence at ages 1–5 years by child birth cohort, by maternal age classes.^a^

Maternal age (years)	APC (%)	95% CI
≤ 24	1.66	0.68 to 2.65
25–29	1.11	0.32 to 1.91
30–34	−0.01	−0.88 to 0.86
≤ 35	0.23	−0.93 to 1.40

a Data from Swedish Cancer Registry, 1960–2004.

**Table 2:** Number of cases^a^ of childhood leukemia at ages 1–5 years and corresponding incidence rates (in parentheses) by maternal birth cohort and age classes.^b^

	Maternal age (years)			
Maternal birth cohort	≤ 24	25–29	30–34	≥ 35
1930–1934	Not available	15 (4.88)	40 (7.47)	28 (11.87)
1935–1939	12 (3.41)	53 (5.75)	31 (6.45)	12 (6.37)
1940–1944	60 (5.24)	61 (5.95)	40 (7.59)	21 (8.76)
1945–1949	74 (5.47)	65 (5.79)	52 (8.27)	31 (9.03)
1950–1954	67 (6.39)	67 (6.94)	51 (7.59)	34 (8.52)
1955–1959	54 (6.34)	73 (7.43)	59 (7.17)	37 (9.09)
1960–1964	51 (6.94)	76 (6.69)	62 (7.31)	14 (6.02)
1965–1969	55 (7.32)	77 (7.18)	37 (7.17)	Not available

a Not included: 152 cases with mothers born before 1930 or after 1969.

b Data from Swedish Cancer Registry, 1960–2004.

**Table 3:** RRs of childhood leukemia at ages 1–5 years and corresponding 95% CIs (in parentheses) by maternal birth cohort and age classes.^a^

	Maternal age (years)				
Maternal birth cohort	≤ 24	25–29	30–34	≤ 35	Test for trend (*p*-value)
1930–1934	Not available	1	1.52 (0.86–2.85)	2.42 (1.31–4.67)	0.004
1935–1939	1	1.67 (0.92–3.29)	1.87 (0.98–3.82)	1.87 (0.82–4.24)	0.095
1940–1944	1	1.13 (0.79–1.62)	1.45 (0.96–2.15)	1.68 (1.00–2.72)	0.020
1945–1949	1	1.06 (0.76–1.48)	1.51 (1.06–2.15)	1.66 (1.07–2.50)	0.006
1950–1954	1	1.09 (0.77–1.53)	1.19 (0.82–1.71)	1.34 (0.87–2.01)	0.148
1955–1959	1	1.17 (0.82–1.67)	1.13 (0.78–1.64)	1.43 (0.94–2.17)	0.121
1960–1964	1	0.96 (0.68–1.38)	1.05 (0.73–1.53)	0.87 (0.46–1.54)	0.871
1965–1969	1	0.98 (0.69–1.39)	0.98 (0.64–1.48)	Not available	0.911

a Data from Swedish Cancer Registry, 1960–2004.
